# Is Antibiotic Prophylaxis Necessary in Mastectomy with Antimicrobial Sutures? A Comparative Analysis

**DOI:** 10.3390/cancers17172892

**Published:** 2025-09-02

**Authors:** Samuli Pajaanti, Carlo M. Oranges, Pietro Giovanni di Summa, Salvatore Giordano

**Affiliations:** 1 Department of Plastic and General Surgery, Turku University Hospital, 21000 Turku, Finland; sajpaa@utu.fi; 2 Department of Clinical Medicine, The University of Turku, 20014 Turku, Finland; 3 Department of Plastic, Reconstructive and Aesthetic Surgery, Geneva University Hospitals, University of Geneva, 1201 Geneva, Switzerland; carlo.oranges@hcuge.ch; 4 Department of Plastic and Hand Surgery, University Hospital of Lausanne (CHUV), University of Lausanne, 1011 Lausanne, Switzerland; pietro.disumma@gmail.com; 5 Department of Surgery, Satasairaala Hospital, 28500 Pori, Finland

**Keywords:** antibacterial, absorbable suture, antibiotic prophylaxis, surgical site infection, mastectomy, breast cancer, breast surgery

## Abstract

Background/Objectives: Surgical site infection (SSI) rates following breast surgical procedures range from 0.8% to 26%. Both prophylactic antibiotics and antimicrobial-coated sutures have been shown to play an important role in reducing these complications. This study aimed to evaluate the impact of antibiotic prophylaxis in mastectomy procedures using triclosan-coated sutures. Methods: This study included 300 consecutive patients who underwent mastectomy for breast cancer over a two-year period, during which triclosan-coated Vicryl Plus sutures were used. Patients were divided into two groups based on the use of antibiotic prophylaxis. The prophylaxis group received 1.5 g cefuroxime intravenously at anesthesia induction (600 mg clindamycin in case of allergy), while the control group received no antibiotics. Endpoints of interest included differences in SSI and specific wound-healing complications at follow-up. Results: There was no significant difference in the overall SSI rates between the two groups: 23.2% in the prophylaxis group vs. 18.8% in the control group [odds ratio (OR): 0.88; 95% confidence interval (CI): 0.69–1.13; vs. OR: 1.16; 95% CI 0.85–1.58; *p* = 0.343]. No adverse drug reactions were observed. *Staphylococcus aureus* was the most isolated microorganism in both groups. Multivariate analysis identified prolonged operative time and hematoma formation as significant predictors of postoperative infection. Conclusions: Antibiotic prophylaxis did not reduce the rate of SSI following mastectomy for breast cancer when triclosan-coated sutures were used. Further high-quality, independent studies are warranted, particularly in breast surgery context.

## Simple Summary

Patients undergoing mastectomy are at risk of developing surgical site infections, which can delay healing and increase healthcare costs. To reduce this risk, antibiotics are often given before surgery, and antibacterial sutures, such as triclosan-coated ones, are also used. However, it remains unclear whether both methods are necessary or if the sutures alone are sufficient. This study explores whether prophylactic antibiotics add any benefit in preventing surgical site infections when triclosan-coated sutures are already used. The goal is to improve patient care by identifying the most effective and safest infection prevention strategy while also reducing unnecessary antibiotic use and its associated resistance risk.

## 1. Introduction

Breast cancer (BC) is the most diagnosed cancer in women worldwide, with an estimated 2.3 million new cases annually, accounting for 11.6% of all cancer diagnoses globally. Despite advancements in breast-conserving techniques, BC still often necessitates radical mastectomy, a procedure linked to high surgical site infection (SSI) rates (up to 41%) and associated complications that significantly impact patient recovery, quality of life, and healthcare costs [1,2,3,4,5,6].

Reducing infection during breast cancer surgery is critical, as SSIs are associated with significantly increased morbidity, mortality, and healthcare costs. Patients with postoperative complications experienced longer delays before starting adjuvant chemotherapy (47 days vs. 41 days, *p* = 0.027) [7]. Another study reported that 39.9% of patients with complications experienced delays in chemotherapy, compared to 21.2% without complications (*p* < 0.001) [8].

Several patient-related risk factors, including high body mass index (BMI), diabetes mellitus, and hypoalbuminemia, are known to be strongly associated with increased postoperative complications, including infections [9,10]. Given the potential for these complications to delay subsequent therapies and negatively affect oncologic outcomes, effective strategies to reduce postoperative morbidity are essential.

Surgical antibiotic prophylaxis is widely used to reduce intraoperative contamination and the risk of surgical site infections (SSIs). However, inappropriate or routine use of antibiotics can lead to adverse effects such as allergic reactions, Clostridium difficile infections, and the development of antimicrobial resistance [11,12,13,14]. In breast cancer surgery, especially total and radical mastectomies often involving drains, the effectiveness of antibiotic prophylaxis remains controversial. Randomized controlled trials (RCTs) have produced conflicting results, with some showing no significant benefit [15,16], while others report a meaningful reduction in SSI rates [17,18]. These inconsistencies highlight the need to better identify patients who would truly benefit from antibiotic prophylaxis to optimize outcomes while minimizing unnecessary exposure. Triclosan-coated polyglactin 910 sutures (Vicryl Plus™, Ethicon Inc., Somerville, NJ, USA) have been introduced as an adjunctive strategy to reduce SSIs by providing local antimicrobial protection [18]. However, a few studies have specifically evaluated the effectiveness of TC-coated sutures in preventing SSIs following breast cancer surgery [19,20,21]. Understanding how to integrate antibiotic prophylaxis with such technologies may improve postoperative infection rates and patient outcomes in breast cancer surgery.

The aim of this study is to evaluate the effect of TC-coated suture material, with or without antibiotic prophylaxis, in mastectomy procedures. Specifically, we seek to quantify the potential benefit of combining prophylactic antibiotics with TC-coated sutures in reducing the incidence of SSIs, with the overarching goal of minimizing postoperative complications, limiting antibiotic resistance, and improving clinical outcomes.

We hypothesize that the use of TC-coated sutures without prophylactic antibiotic administration is comparable to their combined use in reducing the incidence of SSIs in patients undergoing mastectomy for breast cancer.

## 2. Materials and Methods

### 2.1. Study Settings

This retrospective cohort study included consecutive patients diagnosed with operable malignant breast cancer who underwent mastectomy over a two-year period (2015–2017) at Turku University Hospital, Turku, Finland. The study was conducted in accordance with the ethical principles of the World Medical Association Declaration of Helsinki and was approved by the local institutional review board (T145/2017). Owing to the retrospective nature of the study and anonymization of patient data, the requirement for individual informed consent was waived.

### 2.2. Inclusion Criteria

Comprehensive demographic and clinical data were extracted from electronic medical records, including preoperative assessments, oncological histories, and postoperative follow-up data from outpatient visits. A departmental policy change in April 2016 regarding the use of prophylactic antibiotics enabled a comparison between patient cohorts treated with and without surgical antibiotic prophylaxis. Prior to this change, triclosan-coated sutures were almost standard; however, prophylactic antibiotics were not routinely administered, and their use was determined by the surgeon’s judgment based on each patient’s perceived risk of infection.

Inclusion criteria were limited to consecutive female patients who underwent either total or modified radical mastectomy without immediate reconstruction, with or without axillary lymph node dissection, in whom surgical wound closure was performed using TC-coated sutures. Exclusion criteria included prophylactic mastectomy, nipple- or skin-sparing mastectomy, immediate breast reconstruction, and coexisting medical conditions necessitating prolonged antibiotic therapy (e.g., immunosuppression). Patients who underwent additional surgical procedures (e.g., oophorectomy), had documented allergies to TC-coated sutures, or where such sutures were not used were also excluded.

### 2.3. Exposure Definition

Patients were categorized into two groups based on the use of TC-coated sutures, with or without antibiotic prophylaxis. The prophylaxis group received a single intravenous dose of either cefuroxime (1500 mg) or clindamycin (600 mg) at the time of anesthesia induction. The unexposed group did not receive any antibiotic prophylaxis, but TC-coated sutures were used.

Surgical procedures included modified radical mastectomy and total mastectomy with sentinel lymph node biopsy (SNB). Breast tissue was excised en bloc, with anatomical boundaries defined superiorly by the clavicle, medially by the sternum, inferiorly by the inframammary fold, and laterally by the latissimus dorsi, following the fascia of the pectoralis major. Axillary lymph node dissection was performed if SNB revealed metastatic involvement.

**Figure 1:**
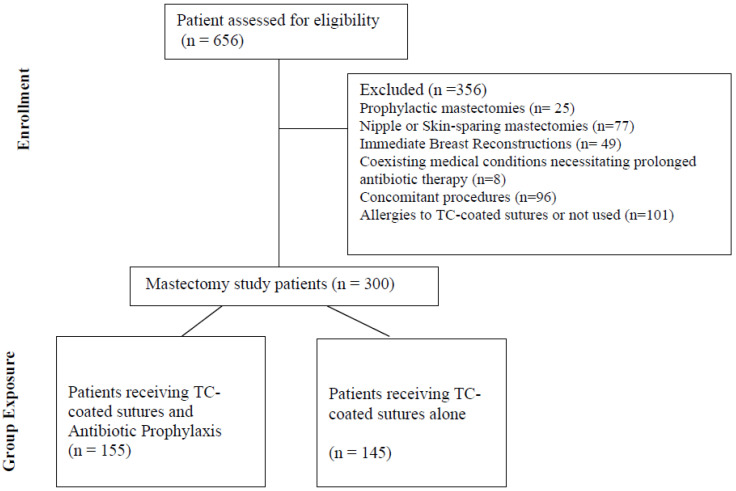
Flowchart diagram of the study inclusion and group exposure.

Antibiotic prophylaxis was administered based on surgeon discretion and assessment of individual patient infection risk prior to April 2016; thereafter, prophylaxis was routinely given to all patients. When indicated, a single intravenous dose of antibiotic was administered within 60 min prior to surgical incision, typically during anesthesia induction. Cefuroxime (1.5 g IV) was used as the first-line agent, while clindamycin (600 mg IV) was reserved for patients with contraindications or documented allergies to cephalosporins. The number of patients receiving each antibiotic regimen is detailed in the flowchart (Figure 1) and the Section 3.

All patients underwent preoperative skin preparation with chlorhexidine-based cleansing, followed by two applications of chlorhexidine solution. All procedures utilized the ultrasonic dissection device SonoSurg^®^ (Olympus Medical Instruments, Tokyo, Japan) or Harmonic FocusTM (Ethicon, Inc., Somerville, NJ, USA). Operation duration was measured from the initial incision to final skin closure.

SNB was performed in patients without clinical or ultrasound evidence of axillary metastases. If nodal metastases were identified preoperatively or intraoperatively, ALND was performed. In cases of reoperation due to positive margins after breast-conserving surgery (BCS), axillary surgery was typically completed during the initial procedure.

Wound closure employed a two-layer suture technique: TC-coated polyglactin 910 sutures (Vicryl Plus™, Ethicon Inc., Somerville, NJ, USA) were used for the dermal layer, and intracutaneous closure was achieved with the V-Loc™ 90 Absorbable Wound Closure Device (Medtronic, Mansfield, MA, USA). One surgical drain was placed per procedure (or two for bilateral mastectomies), with removal recommended on postoperative day six or earlier if output was <80 mL/day. Postoperative follow-up occurred typically 2 to 3 weeks after surgery and as needed afterwards. Overall follow-up was 28.1 ± 7.8 months.

### 2.4. Outcomes

The primary endpoint of this study was to assess the rate of surgical site infections (SSIs) within 30 days following radical mastectomy and to compare the odds of infection between patients receiving triclosan-coated (TC) sutures with antibiotic prophylaxis and those receiving TC sutures alone. An SSI was defined as an infectious process (redness, cellulitis or abscess) requiring at least oral or intravenous antibiotic treatment, with or without surgical intervention. SSIs were identified based on the following criteria: purulent drainage from the surgical site; a positive culture from fluid or tissue obtained from the incision site, accompanied by at least one clinical sign of infection (e.g., pain, localized swelling, erythema, or warmth); or a clinical diagnosis of SSI by the attending physician. A positive microbiological culture alone, without accompanying clinical symptoms, was not sufficient to define an SSI.

Secondary endpoints included differences in complications rates, defined as any complication related to the operated breast, and specific postoperative complications such as skin or fat necrosis, wound dehiscence, hematoma, and seroma.

Wound dehiscence was defined as full-thickness skin separation ≥ 2 cm, with or without infection.

Skin necrosis was defined as clearly demarcated necrotic tissue at the wound edge exceeding 1 cm in width.

Fat necrosis was defined as a palpable, firm mass ≥1 cm in diameter persisting for more than three months postoperatively.

Hematomas and seromas were defined as subcutaneous collections of blood or serous fluid, respectively, requiring percutaneous aspiration or surgical drainage.

Superficial wound infections or redness that were resolved without antibiotic therapy and minor wound dehiscence not requiring specialized wound care were not classified as complications.

All postoperative complications were classified according to Clavien–Dindo system [22]. Additionally, an exploratory analysis was conducted to identify potential predictors of 30-day postoperative SSIs through multivariable logistic regression modeling.

### 2.5. Covariates

Age (mean ± SD): Patient age at the time of surgery, recorded in years as a continuous variable.

BMI (kg/m^2^, mean ± SD): Body mass index calculated using preoperative height and weight, expressed in kg/m^2^.

Any comorbidity: Presence of at least one chronic medical condition documented in the patient’s medical history.

Diabetes: Diagnosis of type 1 or type 2 diabetes mellitus confirmed by medical records or ongoing treatment.

HTA: History of hypertension, defined as documented diagnosis or antihypertensive medication use.

Dyslipidemia: Documented history of abnormal lipid profile or use of lipid-lowering medications.

Obesity: BMI ≥ 30 kg/m^2^, based on WHO classification.

Metabolic syndrome was identified in patients meeting at least three of the following criteria: BMI ≥ 30 kg/m^2^, current treatment for hypertension, diabetes, or dyslipidemia.

Depression: History of clinically diagnosed depression or ongoing pharmacologic treatment at the time of surgery.

Smokers: Patients who had smoked any tobacco products within one month prior to surgery were classified as active smokers.

Operative time (min, mean ± SD): Total duration of the surgical procedure, from incision to closure, recorded in minutes.

Double mastectomy: Patients undergoing bilateral mastectomy during the same operative session.

Mastectomy weight (g, mean ± SD): Total tissue weight removed during mastectomy, recorded in grams from pathology reports.

Estimated blood loss (ml, mean ± SD): Intraoperative blood loss recorded in milliliters, as documented by the anesthesiology team.

Sentinel node biopsy: Whether a sentinel lymph node biopsy was performed during surgery (yes/no).

Axillary dissection: Whether an axillary lymph node dissection was performed (yes/no).

All these data were meticulously collected from electronic medical records and operative reports.

### 2.6. Statistical Analysis

Statistical analyses were performed using IBM SPSS Statistics (version 29; IBM Corp., Armonk, NY, USA). Continuous variables were reported as means ± standard deviations (SD). Continuous variables were assessed for normality using the Shapiro–Wilk test. Normally distributed variables were compared using independent samples *t*-tests, while non-normally distributed variables were analyzed using the Mann–Whitney U test. Categorical variables were compared using chi-square or Fisher’s exact tests, as appropriate. The primary outcome, 30-day postoperative surgical site infection (SSI), was analyzed using binary logistic regression to compare the odds of infection between patients receiving triclosan-coated (TC) sutures with antibiotic prophylaxis and those receiving TC sutures alone. Potential confounders were identified based on clinical relevance and variables with a *p*-value < 0.10 in univariate analysis. These covariates were included in the multivariable logistic regression model to adjust for their potential influence on SSI risk. Odds ratios (ORs) and 95% confidence intervals (CIs) were reported.

All statistical tests were two-tailed, with *p* < 0.05 considered statistically significant.

## 3. Results

A total of 300 consecutive mastectomy patients whose surgical wounds were closed using TC-coated sutures were included in the study (Figure 1): 155 in the prophylaxis group and 145 in the unexposed group. In the TC + antibiotic prophylaxis group, cefuroxime (1.5 g IV) was administered to 134 patients (86.5%), while clindamycin (600 mg IV) was used in 21 patients (13.5%).

The only statistically significant differences in baseline characteristics were the proportion of active smokers (30.2% in the prophylaxis group vs. 19.3% in the control group; *p* = 0.032) and the prevalence of obesity (34.8% vs. 22.7%; *p* = 0.022). No significant differences were observed between the groups in terms of age, body mass index (BMI), comorbidities (including diabetes, hypertension, dyslipidemia, and depression), or BRCA mutation status (Table 1).

When comparing perioperative parameters between the two groups, three variables showed statistically significant differences: estimated blood loss (mean ± SD: 113.5 ± 124.0 mL in the prophylaxis group vs. 76.6 ± 63.9 mL in the control group; *p* = 0.004) and frequency of sentinel lymph node biopsy (51.0% vs. 69.7%; *p* = 0.001). No significant differences were noted in operative time, specimen weight, length of hospital stay, or the number of bilateral mastectomies or axillary lymph node dissections (Table 1).

The overall rate of SSIs was 21.0% (63/300), with 23.3% (36/155) occurring in the prophylaxis group and 18.8% (27/145) in the unexposed group, without reaching statistically significant difference [OR: 0.88; 95% confidence interval (CI): 0.69–1.13; vs. OR: 1.16; 95% CI 0.85–1.58; *p* = 0.343]. There were no statistically significant differences between superficial infections (OR: 1.00; 95% CI: 0.73–1.34; vs. OR: 0.99; 95% CI 0.74–1.33; *p* = 0.980) or deep infections (OR: 1.17; 95% CI: 0.72–1.90; vs. OR: 0.88; 95% CI 0.61–1.26; *p* = 0.507). Similarly, no significant differences were observed in the incidence of other postoperative complications, including seroma, hematoma, wound dehiscence, or skin necrosis between the two groups (Table 2).

Microbiological cultures identified pathogens in 23.8% (15/63) of the confirmed SSI cases. *Staphylococcus aureus* was the most isolated microorganism, found in both groups. There were no significant differences in culture results between the groups (*p* = 0.793; Table 3).

In multivariate logistic regression analysis, hematoma (OR 11.9, 95% CI 5.7–28.6, *p* < 0.001) and operative time (OR 9.2, 95% CI 3.1–10.6, *p* = 0.001) were significant independent predictors of SSI (Table 4).

## 4. Discussion

We found no statistically significant reduction in surgical site infection (SSI) rates associated with adding a single-dose of prophylactic antibiotics to TC-coated sutures. Despite routine implementation of antibiotic prophylaxis, the incidence of SSIs remained comparable between the prophylaxis group (23.3%) and the control group (18.8%, *p* = 0.343). These findings suggest that routine prophylactic antibiotic administration may not confer a measurable advantage in reducing postoperative infections, when TC-coated sutures are also used.

Although retrospective in design, our study represents the largest to date investigating the impact of TC-coated sutures in breast cancer surgery. Previous studies on this topic have been limited in size, including two randomized controlled trials [19,21] and one prospective cohort study [20], with sample sizes ranging from 101 [21] to 190 patients [20]. In one of the randomized trials, antibiotic prophylaxis was administered to only 8 out of 150 patients [19], whereas in the other two studies, all patients received antibiotic prophylaxis [20,21]. Notably, none of these studies demonstrated a statistically significant difference in surgical site infection rates between the intervention and control groups.

In our study, 300 consecutive mastectomy patients were included and divided into two groups: the prophylaxis group (*n* = 155) and the control group (*n* = 145). A notable finding was that the prophylaxis group had a significantly higher proportion of smokers and obese patients, which are both recognized as risk factors for postoperative complications, including SSIs. Smoking impairs wound healing, while obesity is associated with increased surgical risk due to poor tissue perfusion and a higher likelihood of complications such as infection and wound dehiscence [23]. These baseline differences are important, as they may have influenced the study outcomes. However, aside from these factors, no significant differences were observed between the groups in other variables, including the presence of metabolic syndrome. This suggests that the groups were otherwise comparable in terms of risk factors that could affect infection rates.

Regarding perioperative parameters, significant differences were observed between the two groups in estimated blood loss, duration of follow-up, and the frequency of sentinel node biopsies. The prophylaxis group had a higher mean estimated blood loss and underwent fewer sentinel node biopsies (Table 1 and Table 2). Although these factors varied between groups, they did not appear to influence the overall infection rates. The overall incidence of SSIs was 21.0%, with 23.3% of patients in the prophylaxis group and 18.8% in the control group experiencing infections. Notably, no statistically significant differences were found between the groups in terms of total, superficial, or deep infection rates, suggesting that prophylactic antibiotics did not provide additional protection against SSIs in this cohort, when triclosan-coated sutures were used.

Microbiological cultures were obtained in 63 confirmed SSI cases, with *Staphylococcus aureus* identified as the most common pathogen in both groups. This finding aligns with previous studies that have reported *S. aureus* as the predominant organism in breast surgery infections [5,6,16,17,18,24,25]. Despite this, no significant differences in microbiological profiles were observed between the groups (*p* = 0.793, Table 4), further supporting the conclusion that prophylactic antibiotics, when used alongside triclosan-coated sutures, did not significantly influence the microbiological characteristics of SSIs.

Among the variables associated with postoperative infections in this study, a BMI above 25 emerged as a well-documented risk factor [17,23]. Additionally, breast resection weight, also linked to higher SSI incidence, can be considered indirectly related to patient body weight. In our cohort, the optimal cutoff values for predicting postoperative infection were 26.2 kg/m^2^ for BMI and 1019.0 g for breast resection weight [4].

Our findings regarding hematoma formation and prolonged operative time as potential risk factors are consistent with recent studies [26,27]. Specifically, procedures lasting longer than 94.5 min were associated with a higher risk of postoperative infection, regardless of antibiotic prophylaxis use or the application of TC-coated sutures.

Univariate analysis identified several factors significantly associated with SSIs, including BMI, breast resection weight, operative time, hematoma, and seroma formation. These findings agree with the existing literature that highlights these factors as contributors to increased infection risk after mastectomy [23,24,25,26,27]. Operative time and hematoma presence were particularly strongly associated with SSIs, reinforcing the established understanding that prolonged surgeries and the accumulation of blood or fluid in the wound bed can predispose patients to infection.

In multivariate analysis, both operative time and hematoma remained significant independent predictors of SSIs. Operative time was a particularly critical factor (OR: 1.0, *p* = 0.005), while the presence of hematoma significantly increased the risk of infection (OR: 11.1, *p* = 0.045).

Although TC-coated sutures appear to be established in reducing SSIs [18], relatively few studies have specifically addressed the role of prophylactic antibiotics in preventing SSIs following breast cancer surgery. Given the large number of patients affected by breast cancer and its surgical treatments, this represents a significant gap in the literature. A recent systematic review [5], which includes many of the major studies referenced in earlier reviews, suggests that antibiotic prophylaxis may reduce SSIs in patients undergoing non-reconstructive breast cancer surgery. However, considerable heterogeneity exists among the included studies, including variations in antibiotic regimens, dosing protocols, follow-up durations (ranging from 5 to 42 days), and surgical procedures. Furthermore, many of these studies grouped different types of breast surgeries together and included both malignant and non-malignant cases. Since infection risks and associated factors vary across different surgical approaches [16], further research is warranted to specifically evaluate the effectiveness of antibiotic prophylaxis in mastectomy procedures.

Previous prospective trials have generally not supported the routine use of prophylactic antibiotics in breast surgery [15,28,29], while one retrospective study [30] reported favorable outcomes. Notably, that study utilized a collagen–gentamicin sulphate sponge placed in the surgical wound rather than systemic intravenous antibiotics, further complicating direct comparisons across studies.

Despite these methodological differences, the findings of our study align with the broader literature, suggesting limited benefit of prophylactic antibiotics in mastectomy when TC-coated sutures are used. A larger, retrospective study conducted at our own institution involving 1423 patients similarly found no significant difference in overall SSI rates—6.9% in the antibiotic prophylaxis group versus 6.3% in the non-prophylaxis group [31]. However, that study did not account for several critical variables, including distinctions between superficial and deep infections, mastectomy weight, postoperative hematoma, and other surgical site occurrences, which limit the interpretability and clinical relevance of its findings. Our higher infection rate may reflect a combination of factors, including high rates of patient comorbidities (e.g., diabetes, obesity), the high proportion of active smokers (30% in the prophylaxis group), prolonged surgical times, and institutional factors such as varying compliance with infection prevention protocols during the study period. Furthermore, patients who had complications often experienced several types of complications (Table 3). However, most of complications were classified as grade I using the Clavien–Dindo system.

Our results suggest that prophylactic antibiotics do not significantly reduce the incidence of SSIs in patients undergoing mastectomy for breast cancer, when TC-coated sutures are used [18]. Although antibiotic prophylaxis has been shown to reduce SSIs in certain surgical contexts, its benefit in clean procedures such as mastectomy remains debated. Moreover, the routine use of antibiotics is not without risks, as potential adverse effects include drug reactions, increased antimicrobial resistance, and Clostridium difficile infections, which may outweigh the potential benefits [5,11,12,28].

Despite the use of coated sutures and adherence to standard prophylactic measures, the overall SSI rate in our cohort remains above 20%, which, while high, reflects the complexity and comorbidity profile of this patient population. Recognizing this, we are actively exploring additional strategies to further reduce SSI incidence. These include preoperative patient optimization (e.g., improved glycemic control, nutritional support, and smoking cessation), enhanced intraoperative techniques such as glove changes before closure and the use of wound protectors, and postoperative protocols aimed at early detection and management of wound complications. Furthermore, we are piloting the use of negative-pressure wound therapy (NPWT) on closed incisions in high-risk patients as a prophylactic measure. These ongoing efforts reflect a multifaceted approach to infection prevention, beyond suture technology alone. Importantly, the use of a single prophylactic dose, as applied in our protocol, remains in line with current guidelines and is associated with a low risk of promoting bacterial resistance.

The strengths of this study include the homogeneity of the surgical procedures, consistency in surgical technique, and a relatively large sample size compared to previous studies on SSI incidence in mastectomy patients, with or without the use of TC-coated sutures. All included patients met clearly defined criteria and were enrolled consecutively without exclusions. Additionally, most patients had at least one postoperative follow-up visit at the same department where the surgery was performed, and the follow-up period was relatively long.

This study has several limitations. First, although we included a relatively large cohort of mastectomy patients, the retrospective design and absence of randomization introduce the potential for selection bias. Surgeries were performed by 15 different surgeons with varying levels of experience; however, similar dissection techniques and instruments were employed across cases.

There were also notable differences between the study groups. Due to the lack of randomization, it is possible that surgeons selectively prescribed antibiotic prophylaxis to patients perceived as being at higher risk for SSIs. This could have led to an overrepresentation of higher-risk individuals in the prophylaxis group and may explain the higher proportion of smokers and the increased estimated blood loss observed in this group. These factors could also partly account for the higher infection rate seen in the prophylaxis group, despite the difference not reaching statistical significance. One important limitation of this study is the relatively small sample size, which likely contributed to its low statistical power for detecting a significant difference in the primary endpoint, which was any surgical site infection (SSI). Additionally, the small effect size observed suggests that any potential benefit of prophylaxis may be modest and difficult to identify without a substantially larger cohort. The lower number of sentinel node biopsies in the prophylaxis group may suggest that more patients in this group proceeded directly to axillary dissection, potentially due to more advanced disease or other perceived risks. While axillary dissection is known to increase SSI risk, no significant differences in the frequency of axillary dissection were observed between the two groups. The difference in follow-up duration is attributable to the chronological order in which patients were enrolled and is unlikely to have affected the outcomes in a meaningful way.

Additionally, we did not evaluate the impact of neoadjuvant radiotherapy or chemotherapy on infection rates due to the limited number of such cases in our cohort. Future large-scale, prospective randomized controlled trials are needed to better define the role of prophylactic antibiotics in conjunction with triclosan-coated sutures for preventing SSIs in mastectomy patients. These studies should account for other relevant variables such as surgical technique, patient comorbidities, and microbiological profiles.

## 5. Conclusions

The addition of prophylactic antibiotic administration to TC-coated sutures did not significantly reduce the incidence of surgical site infections following mastectomy for breast cancer. Despite the higher rates of smoking and obesity in the prophylaxis group, which are established risk factors for infection, our findings suggest that these interventions may not offer additional benefits in this patient cohort. While the results suggest no significant benefit of prophylactic antibiotics in this context, a comprehensive SSI prevention bundle is likely required, and the findings should be interpreted with caution due to the study’s limited power. Larger, well-powered studies are needed to more definitively assess the role of prophylactic antibiotics in preventing SSIs following mastectomy when antimicrobial sutures are used. Instead, operative time and hematoma formation were identified as the strongest predictors of infection. Our findings also suggest that efforts to optimize surgical efficiency and hemostasis may be more impactful than routine SAP use in reducing postoperative infections. Given the potential risks associated with overuse of antibiotics and antimicrobial resistance, further research is necessary to determine the most effective strategies to reduce SSIs and improve postoperative outcomes in breast cancer surgery.

## Figures and Tables

**Table 1:** Demographics of patients and perioperative parameters.

	TC Suture + Antibiotic Prophylaxis (*n* = 155)	TC Suture Alone (*n* = 145)	*p*-Value
Age (mean ± SD)	67.5 ± 14.6	64.8 ± 15.3	0.108
BMI (kg/m^2^, mean ± SD)	27.5 ± 6.1	26.4 ± 5.4	0.107
Any comorbidity	114 (73.5%)	105 (72.4%)	0.825
Diabetes	15 (9.7%)	15 (10.3%)	0.847
HTA	75 (48.4%)	63 (43.4%)	0.391
Dyslipidemia	33 (21.3%)	26 (18.1%)	0.483
Obesity	54 (34.8%)	33 (22.7%)	0.022
Metabolic syndrome	17 (11.0%)	14 (9.6%)	0.850
Depression	6 (3.9%)	7 (4.8%)	0.684
Smokers	45 (30.2%)	27 (19.3%)	0.032
Operative time (min, mean ± SD)	97.0 ± 27.7	97.9 ± 28.5	0.787
Double mastectomy	16 (10.3%)	8 (5.5%)	0.125
Mastectomy weight (g, mean ± SD)	683.2 ± 393.6	662.8 ± 370.8	0.663
Estimated blood loss (ml, mean ± SD)	113.5 ± 124.0	76.6 ± 63.9	0.004
Sentinel node biopsy	79 (51.0%)	101 (69.7%)	0.001
Axillary dissection	103 (66.5%)	87 (60.0%)	0.247

BMI = Body mass index; HTA = hypertension; TC.

**Table 2:** Postoperative complications.

	TC Suture + Antibiotic Prophylaxis (*n* = 155)	TC Suture Alone (*n* = 145)	*p*-Value
Complications (patients)	41 (26.5%)	37 (25.7%)	0.882
Any infection (SSI)	36 (23.2%)	27 (18.8%)	0.343
*Clavien–Dindo Grade I*			
Superficial infection	26 (16.8%)	24 (16.7%)	0.980
Wound dehiscence	12 (7.7%)	9 (6.2%)	0.603
*Clavien–Dindo Grade II*			
Seroma	113 (72.9%)	100 (69.0%)	0.453
Deep infection	14 (9.0%)	10 (6.9%)	0.507
*Clavien–Dindo Grade IIIa*			
Skin necrosis	4 (2.6%)	0 (0.0%)	0.124
Wound dehiscence	5 (3.2%)	4 (2.7%)	1.000
*Clavien–Dindo Grade IIIb*			
Hematoma	2 (1.3%)	5 (3.5%)	0.268
*Clavien–Dindo Grade IV*	0 (0.0%)	0 (0.0%)	1.000
*Clavien–Dindo Grade V*	0 (0.0%)	0 (0.0%)	1.000

SSI = Surgical site infection.

**Table 3:** Culture results in allocated study groups.

	TC Suture + Antibiotic Prophylaxis (*n* = 155)	TC Suture Alone (*n* = 145)	*p*-Value
*Microrganisms*, *n* (%)	7 (4.5%)	8 (5.5%)	0.793
*Staphylococcus aureus*	3 (1.9%)	5 (3.4%)	0.490
*Staphylococcus epidermidis*	2	0	
*Clostridium perfringes*	0	1	
*Streptococcus betahemolyticus*	0	1	
*Prevotella disiens*	0	1	
*Staphylococcus lugdunensis*	1	2	
*Pseudomonas aeruginosa*	1	0	

**Table 4:** Variables put into a multivariate model for any infection occurrence, adjusted for TC + antibiotic prophylaxis.

	Odds Ratio	95% Confidence Interval	*p*-Value
Hematoma	11.9	5.7–28.6	<0.001
Operative time	9.2	3.1–10.6	0.001
Seroma	4.4	0.9–21.6	0.068
Skin necrosis	3.2	0.6–14.3	0.082
Diabetes	2.9	0.3–37.4	0.358
Age	1.1	1.0–1.1	0.900
Breast resection weight	1.0	1.0–1.2	0.241
Any comorbidity	1.0	0.8–1.1	0.256

## Data Availability

The data supporting the findings of this study are available from the corresponding author upon reasonable request. They are not publicly available due to privacy or ethical restrictions.

## Abbreviations

The following abbreviations are used in this manuscript:

BMI	Body mass index
BRCA	BRCA1 and BRCA2 gene mutations
HTA	Hypertension
IV	Intravenous
SSI	Surgical site infection
TC	Triclosan
